# Supplementary material for: The Evolution and Characterization of the RNA Interference Pathways in Lophotrochozoa
Source: Genome Biol Evol. 2024 May 7;16(5):evae098. doi: 10.1093/gbe/evae098 (PMC11114477; doi:10.1093/gbe/evae098)
Supplement: evae098_Supplementary_Data [file evae098_supplementary_data.zip › Supplementary_materials.zip!/selected_miRNAs/NoGe_selected_miRNAs/mirdeep_pdf_files/NMRB01000024.1_Notospermus_geniculatus_strain_Ushimado_scaffold24,_whole_genome_shotgun_sequence_1007.pdf]

Provisional ID	:	NMRB01000024.1_Notospermus_geniculatus_strain_Ushimado_scaffold24,_whole_genome_shotgun_sequence_1007
Score total	:	19674.7
Score for star read(s)	:	3.9
Score for read counts	:	19666.1
Score for mfe	:	1.7
Score for randfold	:	0
Score for cons. seed	:	3
Total read count	:	38586
Mature read count	:	38450
Loop read count	:	0
Star read count	:	136

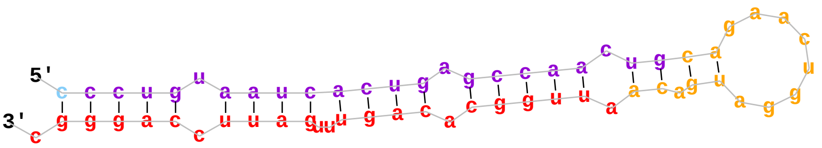

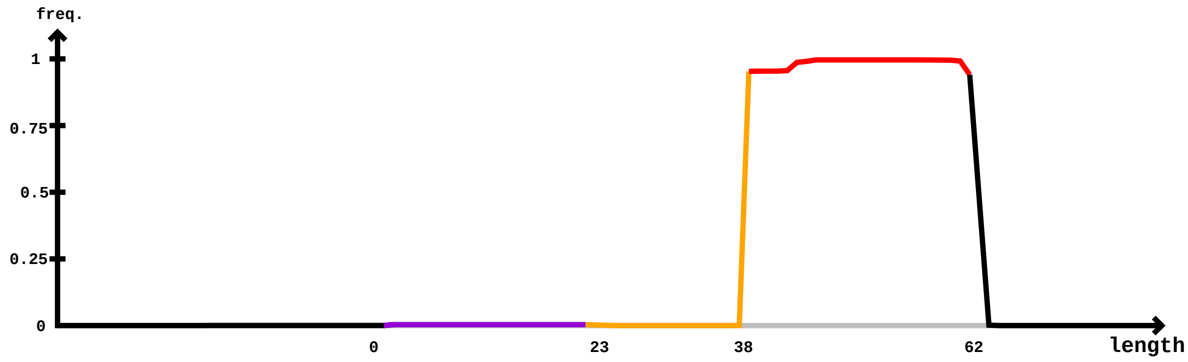

Star **Mature**

```
5'- gaugauggaacaaccacagccgugguuuccgccccuguaaucacugagccaacugcagaacuggaugacaauuggcacaguuugauuccagggcugaaauccacaucaaccua   -3'    obs
    gaugauggaacaaccacagccgugguuuccgccccuguaaucacugagccaacugcagaacuggaugacaauuggcacaguuugauuccagggcugaaauccacaucaaccua          exp
    ((((.((((...(((((....)))))(((.((((.((.((((((((.(((((.((((........)).)).))))).))))..)))).)))))).))).))))))))......   reads      mm      sample
    .................................ccuguaaucacugagccaacu...........................................................   1          0       Fm3
    .................................ccuguaaucacugagccaacug..........................................................   17         0       Fm3
    ......................................................................auuggcacaguuugauucc........................   8          0       Fm3
    ......................................................................auuggcacaguuugauuccag......................   1          0       Fm3
    ......................................................................auuggcacaguuugauuccagg.....................   24         0       Fm3
    ......................................................................auuggcacaguuugauuUcagg.....................   1          1       Fm3
    ......................................................................auuggcacaguuugaCuccaggg....................   1          1       Fm3
    ......................................................................auuggcacaguuugauuUcaggg....................   1          1       Fm3
    ......................................................................auuggcacaguuugauGccaggg....................   1          1       Fm3
    ......................................................................auuggcacaguuugauuGcaggg....................   1          1       Fm3
    ......................................................................auuggcacaguuugauuAcaggg....................   1          1       Fm3
    ......................................................................auuggcacagGuugauuccaggg....................   1          1       Fm3
    ......................................................................auuggcacUguuugauuccaggg....................   1          1       Fm3
    ......................................................................auuggcacaguuugauuccaggg....................   234        0       Fm3
    ......................................................................auuggcacaguuugauuccaggU....................   1          1       Fm3
    ......................................................................auuggcacUguuugauuccagggc...................   2          1       Fm3
    ......................................................................Uuuggcacaguuugauuccagggc...................   1          1       Fm3
    ......................................................................auuggcacaguuugauuccaggUc...................   1          1       Fm3
    ......................................................................auuggcacaguuugauGccagggc...................   2          1       Fm3
    ......................................................................auuggAacaguuugauuccagggc...................   1          1       Fm3
    ......................................................................auuggcaUaguuugauuccagggc...................   1          1       Fm3
    ......................................................................auuggcacaguuugauuccaggAc...................   2          1       Fm3
    ......................................................................auuAgcacaguuugauuccagggc...................   1          1       Fm3
    ......................................................................auuggcacaguuuCauuccagggc...................   1          1       Fm3
    ......................................................................auuggcacaguuugauuccagggA...................   38         1       Fm3
    ......................................................................auuggcacagAuugauuccagggc...................   2          1       Fm3
    ......................................................................auuCgcacaguuugauuccagggc...................   1          1       Fm3
    ......................................................................auuggcacaguuugUuuccagggc...................   1          1       Fm3
    ......................................................................auCggcacaguuugauuccagggc...................   1          1       Fm3
    ......................................................................auuggcacCguuugauuccagggc...................   1          1       Fm3
    ......................................................................auuggcNcaguuugauuccagggc...................   1          1       Fm3
    ......................................................................auugCcacaguuugauuccagggc...................   1          1       Fm3
    ......................................................................auuggcacagGuugauuccagggc...................   2          1       Fm3
```

```
                                 Star                           Mature
gaugauggaacaaccacagccgugguuuccgccccuguaaucacugagccaacugcagaacuggaugacaauuggcacaguuugauuccagggcugaaauccacaucaaccua
......................................................................auuggcacaguuugauucGagggc...................  1     1  Fm3
......................................................................auuggcacaguCugauuccagggc...................  2     1  Fm3
......................................................................auuggcacaguuugauuUcagggc...................  14    1  Fm3
......................................................................auuggcacaguuugauuccaAggc...................  1     1  Fm3
......................................................................auuggcacaguuugauuGcagggc...................  6     1  Fm3
......................................................................auuggcacaguuugauuccaUggc...................  1     1  Fm3
......................................................................auuggcacaguuugauCccagggc...................  7     1  Fm3
......................................................................auuggcacaguuGgauuccagggc...................  1     1  Fm3
......................................................................auuggcacaguuugauuccagggc...................  2070  0  Fm3
......................................................................auuggcacaguuugauAccagggc...................  8     1  Fm3
......................................................................aAuggcacaguuugauuccagggc...................  1     1  Fm3
......................................................................auuggcacagCuugauuccagggc...................  1     1  Fm3
......................................................................auuggcacaguuugauuAcagggc...................  23    1  Fm3
......................................................................auuggcacaUuuugauuccagggc...................  1     1  Fm3
......................................................................auuggcacaguuugauuccagggU...................  59    1  Fm3
......................................................................auuggcaAaguuugauuccagggc...................  1     1  Fm3
......................................................................auuUgcacaguuugauuccagggc...................  2     1  Fm3
......................................................................auuggcacaguuugauucAagggc...................  1     1  Fm3
......................................................................aGuggcacaguuugauuccagggc...................  1     1  Fm3
......................................................................auuggcacaguuugauucUagggc...................  2     1  Fm3
......................................................................auuggcacaguuugauuccagggcu..................  34    0  Fm3
......................................................................auuggcacaguuugauuccagggcA..................  2     1  Fm3
......................................................................auuggcacaguuugauuccagggUu..................  2     1  Fm3
......................................................................auuggUacaguuugauuccagggcu..................  1     1  Fm3
......................................................................auuggcacaguuugauuccagggcug.................  1     0  Fm3
......................................................................auuggcacaguuugauuccagggcuA.................  1     1  Fm3
.......................................................................uuggcacaguuugauuccagggc...................  3     0  Fm3
...........................................................................cacaguuugauuccaggg....................  2     0  Fm3
...........................................................................cacaguuugauuccagggc...................  89    0  Fm3
...........................................................................cacaguuugauuccaggUc...................  2     1  Fm3
...........................................................................cacaguuugauucAagggc...................  1     1  Fm3
...........................................................................cacaguuugaGuccagggc...................  1     1  Fm3
...........................................................................cacaguuugauuccagAgc...................  1     1  Fm3
...........................................................................cacaguuugauuccagCgc...................  1     1  Fm3
...........................................................................cacaguuugauuccaggCc...................  2     1  Fm3
...........................................................................cacaguuugauuccaggAc...................  1     1  Fm3
...........................................................................cacaguuugauuccagggcu..................  7     0  Fm3
............................................................................acaguuugauuccagggc...................  7     0  Fm3
.............................................................................caguuugauuccagggcu..................  23    0  Fm3
.............................................................................caguuugauuccagggcC..................  1     1  Fm3

.................................ccuguaaucacugagccaacu...........................................................  1     0  Mm3
.................................ccuguaaucacugagccGacug..........................................................  1     1  Mm3
.................................ccuguaaucacugagccCacug..........................................................  1     1  Mm3
.................................ccuguaaucacugagccaacug..........................................................  20    0  Mm3
.................................ccuguaaucaAugagccaacug..........................................................  1     1  Mm3
.................................ccuguGaucacugagccaacug..........................................................  1     1  Mm3
.................................ccuguaaucacugagccaacugcagaacu...................................................  1     0  Mm3
.................................ccuguaaucacugagccaacugcagaacuggaugaca...........................................  1     0  Mm3
......................................................................auuggcacaguuugauuc.........................  1     0  Mm3
......................................................................auuggcacaguuugauuccag......................  1     0  Mm3
......................................................................auuggcacaguuugauuccagg.....................  15    0  Mm3
......................................................................auuggcacagAuugauuccagg.....................  1     1  Mm3
......................................................................auAggcacaguuugauuccagg.....................  1     1  Mm3
......................................................................auCggcacaguuugauuccaggg....................  2     1  Mm3
......................................................................auuggcacaguuugauuccaggU....................  2     1  Mm3
......................................................................auuggcacaguuugauuUcaggg....................  4     1  Mm3
......................................................................auuggcacaguuugauuccaggA....................  2     1  Mm3
......................................................................auuggcacagAuugauuccaggg....................  1     1  Mm3
......................................................................auuggcacaguuugauuccaggg....................  220   0  Mm3
......................................................................auuggcacaguuugauCccaggg....................  1     1  Mm3
......................................................................Guggcacaguuugauuccagggc...................  1     1  Mm3
......................................................................auuggcacGguuugauuccagggc...................  1     1  Mm3
......................................................................auuggcacaguuugauuccUgggc...................  1     1  Mm3
......................................................................auuggcacaguuugauuccagggc...................  1266  0  Mm3
......................................................................auuggcacUguuugauuccagggc...................  1     1  Mm3
......................................................................auuggcacaguuugauuUcagggc...................  5     1  Mm3
......................................................................auuggcacaguuugauuAcagggc...................  16    1  Mm3
......................................................................auuggcacaguuugauuccagggU...................  24    1  Mm3
......................................................................auuggcacaguGugauuccagggc...................  1     1  Mm3
```

Star **Mature**

```
gaugauggaacaaccacagccgugguuuccgccccuguaaucacugagccaacugcagaacuggaugacaauuggcacaguuugauuccagggcugaaauccacaucaaccua
......................................................................auuggcacagGuugauuccagggc...................   2     1   Mm3
......................................................................auuggcacaguuugauuGcagggc...................   3     1   Mm3
......................................................................Cuuggcacaguuugauuccagggc...................   2     1   Mm3
......................................................................auuggcacaguuugauGccagggc...................   7     1   Mm3
......................................................................auuggcacaguuuAauuccagggc...................   1     1   Mm3
......................................................................auuggcGcaguuugauuccagggc...................   5     1   Mm3
......................................................................auuggcacagCuugauuccagggc...................   2     1   Mm3
......................................................................auuggcacaguuugauCccagggc...................   1     1   Mm3
......................................................................auuggcacagAuugauuccagggc...................   2     1   Mm3
......................................................................auuggcacaguuugauuccagggA...................   12    1   Mm3
......................................................................auuggcacaguuugauuccagCgc...................   1     1   Mm3
......................................................................Nuuggcacaguuugauuccagggc...................   1     1   Mm3
......................................................................auuAgcacaguuugauuccagggc...................   1     1   Mm3
......................................................................auuggcacaguuugauucUagggc...................   2     1   Mm3
......................................................................auuggcacaguuugauuccagggUu..................   1     1   Mm3
......................................................................auuggcacaguuugauuccagggcA..................   3     1   Mm3
......................................................................auuggcacaguuugauuAcagggcu..................   3     1   Mm3
......................................................................auuggcacGguuugauuccagggcu..................   1     1   Mm3
......................................................................auuggcacaguuugauuccagggAu..................   1     1   Mm3
......................................................................auuggcacaguuugauuccagggcu..................   65    0   Mm3
......................................................................auuggcacaguuugauuccagggcug.................   1     0   Mm3
......................................................................auuggcacaguuugauuccagggcuU.................   5     1   Mm3
........................................................................uggcacaguuugauuccagggc...................   1     0   Mm3
..........................................................................gcacaguuugauuccaggg....................   1     0   Mm3
..........................................................................gcacaguuugauuccaggUc...................   1     1   Mm3
..........................................................................gcaAaguuugauuccagggc...................   1     1   Mm3
..........................................................................gcacaguuugauuccagUgc...................   1     1   Mm3
..........................................................................gcacaguuugauuccagggc...................   28    0   Mm3
..........................................................................gcacaguuugauuccagggcu..................   1     0   Mm3
...........................................................................cacaguuugauuccagggU...................   1     1   Mm3
...........................................................................cacaguuugauuccagggc...................   31    0   Mm3
...........................................................................cacaguuugauuccagggcA..................   2     1   Mm3
...........................................................................cacaguuugauuccagggcu..................   4     0   Mm3
...........................................................................cacaguuugauuccagggcuU.................   3     1   Mm3
............................................................................acaguuugauAccagggc...................   1     1   Mm3
............................................................................acaguuugauuccaggUc...................   1     1   Mm3
............................................................................acaguuugauuccagggc...................   4     0   Mm3
............................................................................acaguuugauuccagggcA..................   1     1   Mm3
............................................................................acaguuugauuccagggcu..................   2     0   Mm3
.............................................................................caguuugauuccagggcA..................   1     1   Mm3
.............................................................................caguuugauuccagggcu..................   17    0   Mm3
.............................................................................caguuugauuccagggcuU.................   3     1   Mm3

......................................................................auuggcacaguuugauuAca.......................   1     1   Fm2
......................................................................auuggcacaguuugauucca.......................   11    0   Fm2
......................................................................auuggcacaguuugauuccag......................   4     0   Fm2
......................................................................auuggcacaguuugauuccagg.....................   11    0   Fm2
......................................................................auuggcacaguuugauuccaggU....................   5     1   Fm2
......................................................................auuggcacaguuugauuAcaggg....................   1     1   Fm2
......................................................................auuggcacaguuugauuccaggg....................   129   0   Fm2
......................................................................auuggcacaguuugauuGcaggg....................   2     1   Fm2
......................................................................auuggcacUguuugauuccaggg....................   1     1   Fm2
......................................................................auuggcacaguuugauuccagggU...................   14    1   Fm2
......................................................................auuggcacaguuugauAccagggc...................   9     1   Fm2
......................................................................auuggcacaguuugauuccagUgc...................   2     1   Fm2
......................................................................aGuggcacaguuugauuccagggc...................   1     1   Fm2
......................................................................auuggcacaguuugauuAcagggc...................   55    1   Fm2
......................................................................auuggcUcaguuugauuccagggc...................   2     1   Fm2
......................................................................auuggcaUaguuugauuccagggc...................   3     1   Fm2
......................................................................auuggcacaguuugauuccagggc...................   4659  0   Fm2
......................................................................auuggNacaguuugauuccagggc...................   1     1   Fm2
......................................................................auuggcacaguuugauuGcagggc...................   28    1   Fm2
......................................................................auuggcacagAuugauuccagggc...................   6     1   Fm2
......................................................................auuggcacaguuugauuccaggAc...................   1     1   Fm2
......................................................................auuggcacGguuugauuccagggc...................   3     1   Fm2
......................................................................auugUcacaguuugauuccagggc...................   1     1   Fm2
......................................................................auuggcacaguuugauGccagggc...................   15    1   Fm2
......................................................................auuggcacaguuugauuUcagggc...................   41    1   Fm2
......................................................................auGggcacaguuugauuccagggc...................   2     1   Fm2
......................................................................Guuggcacaguuugauuccagggc...................   3     1   Fm2
```

Star **Mature**

gaugauggaacaaccacagccgugguuuccgccccuguaaucacugagccaacugcagaacuggaugacaauuggcacaguuugauuccagggcugaaauccacaucaaccua			
....auuggcacUguuugauuccagggc...................	3	1	Fm2
....auuggcacaguuugauuccagCgc...................	1	1	Fm2
....auuggcacaguGugauuccagggc...................	1	1	Fm2
....auuUgcacaguuugauuccagggc...................	2	1	Fm2
....auCggcacaguuugauuccagggc...................	1	1	Fm2
....Uuuggcacaguuugauuccagggc...................	3	1	Fm2
....auuggcacagGuugauuccagggc...................	5	1	Fm2
....auuggcacaguuugauCccagggc...................	13	1	Fm2
....auuCgcacaguuugauuccagggc...................	1	1	Fm2
....auuggcacaguuugauuccaUggc...................	2	1	Fm2
....auuggcacaguuugauuccaggUc...................	1	1	Fm2
....auuggcacagCuugauuccagggc...................	3	1	Fm2
....Nuuggcacaguuugauuccagggc...................	2	1	Fm2
....auuAgcacaguuugauuccagggc...................	1	1	Fm2
....auuggcacaguuugauuccagggA...................	19	1	Fm2
....auuggcacaguuugauuccGgggc...................	3	1	Fm2
....auuggUacaguuugauuccagggc...................	1	1	Fm2
....auuggcacaguuugauuccagAgc...................	2	1	Fm2
....auuggcacaUuuugauuccagggc...................	2	1	Fm2
....auuggcacaguuugauuccagggcA..................	18	1	Fm2
....auuggcacaguuugaCuccagggcu..................	1	1	Fm2
....auuggcacaguuugauuccagggcu..................	55	0	Fm2
....auuggcacagGuugauuccagggcu..................	1	1	Fm2
....auuggcacaguuugauAccagggcu..................	1	1	Fm2
....auuggcacaguuugauuccagggcG..................	4	1	Fm2
....auuggcacaguuugauuGcagggcu..................	1	1	Fm2
....auuggcacaguuugauuUcagggcu..................	1	1	Fm2
....uuggcacaguuugauuccagggc...................	5	0	Fm2
....cacaguuugauuccagggc...................	95	0	Fm2
....cacaguuugauuccaggCc...................	1	1	Fm2
....cacaguuugaAuccagggc...................	1	1	Fm2
....cacaguuugauuccagggcu..................	4	0	Fm2
....cacaguuugauuccagggcG..................	2	1	Fm2
....acaguuugauuccagggc...................	7	0	Fm2
....acaguuugauuccagggcu..................	2	0	Fm2
....caguuugauuccagggcu..................	17	0	Fm2
....caguuugauuccagggcG..................	2	1	Fm2
.................................ccuguaaucacugagccaacug..	15	0	Fm1
.................................ccuguaaucacugagccaacugc...	2	0	Fm1
....Cauuggcacaguuugauuccaggg....................	3	1	Fm1
....auuggcacaguuugauucc........................	2	0	Fm1
....auuggcacaguuugauucca.......................	6	0	Fm1
....auuggcacaguuugauuccaU.......................	1	1	Fm1
....auuggcacaguuugauuccagg.....................	19	0	Fm1
....auuggcacaguuugauGccagg.....................	1	1	Fm1
....auuAgcacaguuugauuccaggg....................	1	1	Fm1
....auuggcacagAuugauuccaggg....................	1	1	Fm1
....auuggcacaguuugauuUcaggg....................	4	1	Fm1
....auuggcacaguuugauuGcaggg....................	1	1	Fm1
....auuggcacaguuugauuAcaggg....................	2	1	Fm1
....auuggcacaguuugauAccaggg....................	3	1	Fm1
....auuggcacaguuugauuccCggg....................	1	1	Fm1
....auuggcacaguuugauGccaggg....................	1	1	Fm1
....auuggcacaguuugauuccaggg....................	395	0	Fm1
....auuggcacaguuugauuccaggU....................	9	1	Fm1
....auuggcacaguuugauuccGggg....................	1	1	Fm1
....auuggcacaguuugauuccGgggc...................	1	1	Fm1
....auuggcacaguuugauuccagAgc...................	2	1	Fm1
....auuggNacaguuugauuccagggc...................	1	1	Fm1
....auuggcacaguuugauuccagggc...................	7782	0	Fm1
....auuggcacaAuuugauuccagggc...................	1	1	Fm1
....auuggcacaguuugUuuccagggc...................	1	1	Fm1
....auuggcacagAuugauuccagggc...................	9	1	Fm1
....auuggcacaguuugauuccCgggc...................	1	1	Fm1
....auuggcacaguuugauGccagggc...................	19	1	Fm1
....auuggcacaguuugauuccaggUc...................	2	1	Fm1
....auuggcacaguuAgauuccagggc...................	1	1	Fm1
....auuggcaGaguuugauuccagggc...................	2	1	Fm1
....auugCcacaguuugauuccagggc...................	1	1	Fm1

Star **Mature**

```
gaugauggaacaaccacagccgugguuuccgccccuguaaucacugagccaacugcagaacuggaugacaauuggcacaguuugauuccagggcugaaauccacaucaaccua
......................................................................auuggcacaUuuugauuccagggc...................   1     1   Fm1
......................................................................auuggcacaguuugauuccagUgc...................   1     1   Fm1
......................................................................auugUcacaguuugauuccagggc...................   3     1   Fm1
......................................................................auuggcacUguuugauuccagggc...................   13    1   Fm1
......................................................................auGggcacaguuugauuccagggc...................   4     1   Fm1
......................................................................auuggcacGguuugauuccagggc...................   2     1   Fm1
......................................................................auuggUacaguuugauuccagggc...................   2     1   Fm1
......................................................................Nuuggcacaguuugauuccagggc...................   2     1   Fm1
......................................................................aCuggcacaguuugauuccagggc...................   1     1   Fm1
......................................................................auuggcacaguuugauucAagggc...................   1     1   Fm1
......................................................................auuggcaUaguuugauuccagggc...................   2     1   Fm1
......................................................................auuggcacaguuuUauuccagggc...................   1     1   Fm1
......................................................................auuggcacagCuugauuccagggc...................   9     1   Fm1
......................................................................Uuuggcacaguuugauuccagggc...................   6     1   Fm1
......................................................................auugAcacaguuugauuccagggc...................   3     1   Fm1
......................................................................auuggcacaguuugauuGcagggc...................   29    1   Fm1
......................................................................auuggcacaguuugauAccagggc...................   17    1   Fm1
......................................................................auuggcacagGuugauuccagggc...................   9     1   Fm1
......................................................................auuUgcacaguuugauuccagggc...................   1     1   Fm1
......................................................................auuggcacaguuugauuccagggA...................   16    1   Fm1
......................................................................aAuggcacaguuugauuccagggc...................   2     1   Fm1
......................................................................auuggcacaguuugauCccagggc...................   26    1   Fm1
......................................................................auuggcGcaguuugauuccagggc...................   5     1   Fm1
......................................................................auuggcUcaguuugauuccagggc...................   4     1   Fm1
......................................................................auuggcacaguuugauuUcagggc...................   37    1   Fm1
......................................................................auuggcacCguuugauuccagggc...................   1     1   Fm1
......................................................................auuggcacaguuugGuuccagggc...................   1     1   Fm1
......................................................................auuggcacaguuugaCuccagggc...................   2     1   Fm1
......................................................................auuggcacaguuugauuccaggAc...................   3     1   Fm1
......................................................................auuggcacaguuugauuAcagggc...................   66    1   Fm1
......................................................................Guuggcacaguuugauuccagggc...................   8     1   Fm1
......................................................................auuggcacaguuugauuccagggU...................   91    1   Fm1
......................................................................auuggcacaguuugauuccagggG...................   1     1   Fm1
......................................................................auCggcacaguuugauuccagggc...................   2     1   Fm1
......................................................................auuggcacaguuugauuUcagggcu..................   2     1   Fm1
......................................................................auuggcacaguuugauuccagggUu..................   1     1   Fm1
......................................................................auuggcacaguuugauuccagggcu..................   204   0   Fm1
......................................................................auuggcacaguuugauAccagggcu..................   1     1   Fm1
......................................................................auuggcacagAuugauuccagggcu..................   2     1   Fm1
......................................................................auuggcacaguuugauuccagggcG..................   7     1   Fm1
......................................................................auuggcacaguuugauucUagggcu..................   1     1   Fm1
......................................................................auuggcacaguuugauuccagggcA..................   9     1   Fm1
......................................................................Cuuggcacaguuugauuccagggcu..................   1     1   Fm1
......................................................................auuggcacaguuugauuccagggcCg.................   1     1   Fm1
......................................................................auuggcacaguuugauuccagggcuU.................   6     1   Fm1
.......................................................................uuggcacaguuugauuccagggc...................   1     0   Fm1
..........................................................................gcacaguuugauuccagggc...................   4     0   Fm1
...........................................................................cacaguuugauuccaggg....................   5     0   Fm1
...........................................................................cacaguuugauuccaggA....................   1     1   Fm1
...........................................................................cacaguuugauuccaggUc...................   1     1   Fm1
...........................................................................cacaguuugauuccagggc...................   52    0   Fm1
...........................................................................cacaguuugauuccagggcu..................   2     0   Fm1
............................................................................acaguuugauuccagggc...................   10    0   Fm1
............................................................................acaguuugauuccagggU...................   1     1   Fm1
............................................................................acaguuugauuccaggCc...................   1     1   Fm1
............................................................................acaguuugauuccagggcu..................   3     0   Fm1
............................................................................acaguuugauuccagggcuU.................   2     1   Fm1
.............................................................................caguuugauuccagggcu..................   19    0   Fm1
.............................................................................caguuugauuccagggcuU.................   4     1   Fm1

..............cacagccgugguuuccgcc................................................................................   8     0   Mm1
.................................ccuguaaucacugagccaacug..........................................................   19    0   Mm1
......................................................................auuggcacaguuugauuccag......................   3     0   Mm1
......................................................................auuggcacaguuugauuccagg.....................   21    0   Mm1
......................................................................auuggcacagCuugauuccagg.....................   1     1   Mm1
......................................................................auuggcUcaguuugauuccaggg....................   1     1   Mm1
......................................................................auuggcacaUuuugauuccaggg....................   1     1   Mm1
......................................................................auuggcacaguuugauuGcaggg....................   4     1   Mm1
......................................................................auuggcacaguuugauuAcaggg....................   2     1   Mm1
......................................................................auGggcacaguuugauuccaggg....................   1     1   Mm1
```

```
                               Star                            Mature
gaugauggaacaaccacagccgugguuuccgccccuguaaucacugagccaacugcagaacuggaugacaauuggcacaguuugauuccagggcugaaauccacaucaaccua
......................................................................auuggcacaguuugauuUcaggg....................    1     1    Mm1
......................................................................auuggcacaguuugauAccaggg....................    3     1    Mm1
......................................................................auuggcacagAuugauuccaggg....................    1     1    Mm1
......................................................................auuggcacaguuugauuccaggA....................    5     1    Mm1
......................................................................auuggcacaguuugauuccaggg....................    370   0    Mm1
......................................................................auuggcacagAuugauuccagggc...................    13    1    Mm1
......................................................................aGuggcacaguuugauuccagggc...................    2     1    Mm1
......................................................................auuggcacaguuugauCccagggc...................    20    1    Mm1
......................................................................Cuuggcacaguuugauuccagggc...................    1     1    Mm1
......................................................................auuggcacCguuugauuccagggc...................    1     1    Mm1
......................................................................auuggcacaguuugauuccaggAc...................    1     1    Mm1
......................................................................auuggcacaguuugauuccagggc...................    5895  0    Mm1
......................................................................auugCcacaguuugauuccagggc...................    1     1    Mm1
......................................................................auuggcacaguuugauuccagggU...................    12    1    Mm1
......................................................................Uuuggcacaguuugauuccagggc...................    6     1    Mm1
......................................................................Nuuggcacaguuugauuccagggc...................    2     1    Mm1
......................................................................auuCgcacaguuugauuccagggc...................    1     1    Mm1
......................................................................auuggUacaguuugauuccagggc...................    1     1    Mm1
......................................................................auuggcacaguuugauuGcagggc...................    23    1    Mm1
......................................................................auuggcacaguAugauuccagggc...................    1     1    Mm1
......................................................................aAuggcacaguuugauuccagggc...................    5     1    Mm1
......................................................................auuggcacaguuugauAccagggc...................    14    1    Mm1
......................................................................auuggcacaguuugauGccagggc...................    17    1    Mm1
......................................................................auuggcacaguuugauucGagggc...................    1     1    Mm1
......................................................................auuggcaUaguuugauuccagggc...................    1     1    Mm1
......................................................................auuggcacaguuugauuccGgggc...................    2     1    Mm1
......................................................................auuggcNcaguuugauuccagggc...................    1     1    Mm1
......................................................................auuggcacaguuugGuuccagggc...................    1     1    Mm1
......................................................................auuggcacagCuugauuccagggc...................    5     1    Mm1
......................................................................auuggcacaguuugauucUagggc...................    1     1    Mm1
......................................................................Guuggcacaguuugauuccagggc...................    8     1    Mm1
......................................................................auuggcacagGuugauuccagggc...................    7     1    Mm1
......................................................................auuggcacaguuugauuccagAgc...................    1     1    Mm1
......................................................................auuggcacaguuugaCuccagggc...................    2     1    Mm1
......................................................................auuggcacaguuugauuccagggA...................    36    1    Mm1
......................................................................auuggcGcaguuugauuccagggc...................    1     1    Mm1
......................................................................auuggcacaguuugCuuccagggc...................    2     1    Mm1
......................................................................auugUcacaguuugauuccagggc...................    1     1    Mm1
......................................................................auuggcacaguuugUuuccagggc...................    3     1    Mm1
......................................................................auuggcacaguuugauuUcagggc...................    29    1    Mm1
......................................................................auAggcacaguuugauuccagggc...................    2     1    Mm1
......................................................................auCggcacaguuugauuccagggc...................    5     1    Mm1
......................................................................auuggcacUguuugauuccagggc...................    8     1    Mm1
......................................................................auuggcacaguuugauucAagggc...................    1     1    Mm1
......................................................................auuggcacaguuugauuAcagggc...................    49    1    Mm1
......................................................................auuggcacaguuugauuUcagggcu..................    1     1    Mm1
......................................................................auuggcacaguuugauuccagggcA..................    11    1    Mm1
......................................................................auuggcacaguuugauuccagggcu..................    74    0    Mm1
......................................................................auuggcacaguuugauuccagggcug.................    2     0    Mm1
......................................................................auuggcacaguuugauuccagggcuU.................    4     1    Mm1
.......................................................................uuggcacaguuugauuccaggg....................    1     0    Mm1
.......................................................................uuggcacaguuugauuccagggc...................    1     0    Mm1
.......................................................................uuggcacaguuugUuuccagggc...................    1     1    Mm1
.........................................................................ggcacaguuugauuccagggc...................    3     0    Mm1
..........................................................................gcacaguuugauuccagggc...................    26    0    Mm1
..........................................................................Acacaguuugauuccagggc...................    1     1    Mm1
..........................................................................gcacaguuugauuccagggcu..................    2     0    Mm1
...........................................................................cacaguuugauuccaggg....................    2     0    Mm1
...........................................................................cacaguuugauuccagggU...................    1     1    Mm1
...........................................................................cacagCuugauuccagggc...................    1     1    Mm1
...........................................................................cacaguuugaGuccagggc...................    2     1    Mm1
...........................................................................cacaguuugaCuccagggc...................    1     1    Mm1
...........................................................................Uacaguuugauuccagggc...................    1     1    Mm1
...........................................................................cacaguuugauuccagUgc...................    1     1    Mm1
...........................................................................cacaguuugauuccaggCc...................    3     1    Mm1
...........................................................................cacaguGugauuccagggc...................    1     1    Mm1
...........................................................................cacaguuugauuccagAgc...................    3     1    Mm1
...........................................................................cacaguuugauuccaggAc...................    6     1    Mm1
...........................................................................cacaguuugaAuccagggc...................    2     1    Mm1
...........................................................................cacaguuugauuccagggc...................    666   0    Mm1
```

Star Mature

```
gaugauggaacaaccacagccgugguuuccgccccuguaaucacugagccaacugcagaacuggaugacaauuggcacaguuugauuccagggcugaaauccacaucaaccua
...........................................................................cacaguuugauuccagggcu..................  41     0  Mm1
............................................................................acaguuugauuccaggCc...................  1      1  Mm1
............................................................................acaguuugauuccagggA...................  1      1  Mm1
............................................................................acaguuugauuccagggc...................  86     0  Mm1
.............................................................................caguuugauuccagggcA..................  5      1  Mm1
.............................................................................caguuugauuccagggcG..................  2      1  Mm1
.............................................................................caguuugauuccagggcu..................  66     0  Mm1
.............................................................................caguuugauuccagggcuU.................  3      1  Mm1

.................................ccuguaaucacugagccaacu...........................................................  6      0  Mm2
.................................ccuguaaucacugagccaacug..........................................................  50     0  Mm2
.................................ccuguaaucacugagccaacGg..........................................................  1      1  Mm2
.......................................................cagaacuggaugacaauuggcaca..................................  2      0  Mm2
.....................................................................Cauuggcacaguuugauuccagg.....................  1      1  Mm2
.....................................................................Cauuggcacaguuugauuccagggc...................  3      1  Mm2
......................................................................auuggcacaguuugauucc........................  2      0  Mm2
......................................................................auuggcacaguuugauucca.......................  1      0  Mm2
......................................................................auuggcacaguuugauuGcagg.....................  1      1  Mm2
......................................................................auuggcacaguuugauuccagg.....................  19     0  Mm2
......................................................................auuggcacaguuugauuccaggg....................  525    0  Mm2
......................................................................auuggcacagCuugauuccaggg....................  1      1  Mm2
......................................................................auuggcacaguuugauuccaggU....................  1      1  Mm2
......................................................................auuggcacaguuugauuGcaggg....................  4      1  Mm2
......................................................................auuggcacaguuugauuccaggA....................  1      1  Mm2
......................................................................auuggcacaguuugauuAcaggg....................  3      1  Mm2
......................................................................auuggcacaguuugauCccaggg....................  3      1  Mm2
......................................................................auuggcacaguuugauuccaggC....................  4      1  Mm2
......................................................................auuggcacaguuugauAccaggg....................  1      1  Mm2
......................................................................auuggcacagGuugauuccaggg....................  1      1  Mm2
......................................................................auuggcacagAuugauuccaggg....................  3      1  Mm2
......................................................................auuggcacaguuugauGccaggg....................  1      1  Mm2
......................................................................auuggcacUguuugauuccaggg....................  1      1  Mm2
......................................................................auuggcacaguuugGuuccaggg....................  1      1  Mm2
......................................................................auuggcacaguuugauuUcaggg....................  1      1  Mm2
......................................................................Uuggcacaguuugauuccagggc...................  11     1  Mm2
......................................................................auuAgcacaguuugauuccagggc...................  1      1  Mm2
......................................................................auuggcacaguuugauGccagggc...................  18     1  Mm2
......................................................................auuggNacaguuugauuccagggc...................  2      1  Mm2
......................................................................auuggcacaguuuUauuccagggc...................  3      1  Mm2
......................................................................auuggcacaguuugauuccagggU...................  72     1  Mm2
......................................................................auAggcacaguuugauuccagggc...................  2      1  Mm2
......................................................................auuggcacGguuugauuccagggc...................  1      1  Mm2
......................................................................auuggcacaguuugauuccaggAc...................  2      1  Mm2
......................................................................auuggcacaguuugauAccagggc...................  29     1  Mm2
......................................................................Cuuggcacaguuugauuccagggc...................  1      1  Mm2
......................................................................auuggcacaguuugaCuccagggc...................  2      1  Mm2
......................................................................auuggcacaguuugauuccagggG...................  1      1  Mm2
......................................................................Guuggcacaguuugauuccagggc...................  10     1  Mm2
......................................................................aCuggcacaguuugauuccagggc...................  3      1  Mm2
......................................................................auuggcacaguuugauuccaggCc...................  3      1  Mm2
......................................................................auuggcacaguuugauucAagggc...................  1      1  Mm2
......................................................................auuggcacagAuugauuccagggc...................  21     1  Mm2
......................................................................auuggcacaguuugauuAcagggc...................  76     1  Mm2
......................................................................auuggcacaguuugauucUagggc...................  6      1  Mm2
......................................................................auuggcacaguuugauuccagggc...................  10396  0  Mm2
......................................................................auuggAacaguuugauuccagggc...................  3      1  Mm2
......................................................................auugUcacaguuugauuccagggc...................  2      1  Mm2
......................................................................auuCgcacaguuugauuccagggc...................  2      1  Mm2
......................................................................auuUgcacaguuugauuccagggc...................  1      1  Mm2
......................................................................aAuggcacaguuugauuccagggc...................  8      1  Mm2
......................................................................auuggcCcaguuugauuccagggc...................  1      1  Mm2
......................................................................auuggcacaguuugaAuccagggc...................  2      1  Mm2
......................................................................auuggcacagCuugauuccagggc...................  16     1  Mm2
......................................................................auuggcaUaguuugauuccagggc...................  2      1  Mm2
......................................................................auuggcNcaguuugauuccagggc...................  1      1  Mm2
......................................................................auuggcacUguuugauuccagggc...................  14     1  Mm2
......................................................................Nuuggcacaguuugauuccagggc...................  2      1  Mm2
......................................................................auuggcacaguuGgauuccagggc...................  1      1  Mm2
......................................................................auuggcacaguuugauuGcagggc...................  48     1  Mm2
......................................................................auuggcacaguuugauuccaAggc...................  3      1  Mm2
```

```
                                 Star                                Mature
gaugauggaacaaccacagccgugguuuccgccccuguaaucacugagccaacugcagaacuggaugacaauuggcacaguuugauuccagggcugaaauccacaucaaccua
......................................................................auuggcacagGuugauuccagggc...................  12   1  Mm2
......................................................................auuggcacaguuCgauuccagggc...................  1    1  Mm2
......................................................................auuggUacaguuugauuccagggc...................  2    1  Mm2
......................................................................auuggcacaguAugauuccagggc...................  1    1  Mm2
......................................................................auuggcacaguuugUuuccagggc...................  2    1  Mm2
......................................................................auGggcacaguuugauuccagggc...................  3    1  Mm2
......................................................................auuggcacaguuuCauuccagggc...................  1    1  Mm2
......................................................................auuggcacaUuuugauuccagggc...................  4    1  Mm2
......................................................................auuggcacaAuuugauuccagggc...................  1    1  Mm2
......................................................................auuggcacaguuugauuUcagggc...................  56   1  Mm2
......................................................................auuggcacaguuugauuccaggUc...................  2    1  Mm2
......................................................................auuggcGcaguuugauuccagggc...................  13   1  Mm2
......................................................................auugAcacaguuugauuccagggc...................  2    1  Mm2
......................................................................auuggcacaguuugauuccagggA...................  37   1  Mm2
......................................................................auuggcacaguuugauCccagggc...................  28   1  Mm2
......................................................................auuggcacaguuugauuccagggcC..................  1    1  Mm2
......................................................................auuggcacaguuugauuccagggcG..................  5    1  Mm2
......................................................................auuggcacaguuugauuccagggAu..................  3    1  Mm2
......................................................................auuggcacaguuugauuGcagggcu..................  2    1  Mm2
......................................................................auuggcacaguuugauuccagggcA..................  6    1  Mm2
......................................................................auCggcacaguuugauuccagggcu..................  1    1  Mm2
......................................................................auuggcacaguuugauAccagggcu..................  2    1  Mm2
......................................................................auuggcacaguuugauuccagggUu..................  4    1  Mm2
......................................................................auuggcacaguuugauuccagggcu..................  244  0  Mm2
......................................................................auuggcacaguuugauuAcagggcu..................  2    1  Mm2
......................................................................auuggcacaguuugauuccagggcuA.................  1    1  Mm2
......................................................................auuggcacaguuugauuccagggcuU.................  13   1  Mm2
......................................................................auuggcacaguuugauuccagggcug.................  3    0  Mm2
.......................................................................uuggcacaguuugauuccagggc...................  8    0  Mm2
........................................................................uggcacaguuugauuccagggc...................  3    0  Mm2
.........................................................................ggcacaguuugauuccagggc...................  3    0  Mm2
..........................................................................Acacaguuugauuccaggg....................  1    1  Mm2
..........................................................................Acacaguuugauuccagggc...................  1    1  Mm2
..........................................................................gcacaguuugauuccagUgc...................  1    1  Mm2
..........................................................................gcacaguuugauuccagggc...................  8    0  Mm2
...........................................................................cacaguuugauuccaggUc...................  3    1  Mm2
...........................................................................cacaguuugauuccagggc...................  116  0  Mm2
...........................................................................cacaguuugauuccaggCc...................  2    1  Mm2
...........................................................................cacaguuugauuccagggcu..................  17   0  Mm2
...........................................................................cacaguuugauuccagggcG..................  1    1  Mm2
...........................................................................cacaguuugauuccagggcuU.................  1    1  Mm2
............................................................................acaguuugauuccagggU...................  1    1  Mm2
............................................................................acaguuugauuccagggc...................  20   0  Mm2
............................................................................acaguuugauuccagggcu..................  2    0  Mm2
.............................................................................caguuugauuccagggcA..................  1    1  Mm2
.............................................................................caguuugauuccagggcu..................  40   0  Mm2
.............................................................................caguuugauuccagggcG..................  2    1  Mm2
.............................................................................caguuugauuccagggcC..................  1    1  Mm2
.............................................................................caguuugauuccagggcuU.................  4    1  Mm2
.............................................................................caguuugauuccagggcug.................  1    0  Mm2
```
